# Diagnosing interstitial lung disease by multidisciplinary discussion: A review

**DOI:** 10.3389/fmed.2022.1017501

**Published:** 2022-09-21

**Authors:** Laura M. Glenn, Lauren K. Troy, Tamera J. Corte

**Affiliations:** ^1^Department of Respiratory and Sleep Medicine, Royal Prince Alfred Hospital, Camperdown, NSW, Australia; ^2^The University of Sydney School of Medicine (Central Clinical School), Sydney, NSW, Australia; ^3^National Health and Medical Research Council (NHMRC) Centre of Research Excellence in Pulmonary Fibrosis, Camperdown, NSW, Australia

**Keywords:** interstitial lung disease (ILD), multidisciplinary meeting (MDM), diagnosis, connective tissue disease (CTD), progressive pulmonary fibrosis

## Abstract

The multidisciplinary meeting (MDM) has been endorsed in current international consensus guidelines as the gold standard method for diagnosis of interstitial lung disease (ILD). In the absence of an accurate and reliable diagnostic test, the agreement between multidisciplinary meetings has been used as a surrogate marker for diagnostic accuracy. Although the ILD MDM has been shown to improve inter-clinician agreement on ILD diagnosis, result in a change in diagnosis in a significant proportion of patients and reduce unclassifiable diagnoses, the ideal form for an ILD MDM remains unclear, with constitution and processes of ILD MDMs varying greatly around the world. It is likely that this variation of practice contributes to the lack of agreement seen between MDMs, as well as suboptimal diagnostic accuracy. A recent Delphi study has confirmed the essential components required for the operation of an ILD MDM. The ILD MDM is a changing entity, as it incorporates new diagnostic tests and genetic markers, while also adapting in its form in response to the obstacles of the COVID-19 pandemic. The aim of this review was to evaluate the current evidence regarding ILD MDM and their role in the diagnosis of ILD, the practice of ILD MDM around the world, approaches to ILD MDM standardization and future directions to improve diagnostic accuracy in ILD.

## Introduction

Interstitial lung disease (ILD) refers to a diverse group of disorders characterized by varying degrees of inflammation and/or fibrosis of the lung parenchyma (1). Due to multiple factors including atypical or overlapping patterns on radiology or histopathology, disease course heterogeneity and rarity of some diseases, the diagnosis of ILD is frequently challenging for clinicians. Despite extensive evaluation, the diagnosis remains unclassifiable in up to 10–20% of cases (2, 3).

Multidisciplinary discussion has been considered the gold standard method for diagnosis of ILDs for the past two decades. The ILD multidisciplinary meeting (MDM) involves dynamic discussion between different subspecialists whereby all available case details are carefully reviewed, and a consensus diagnosis reached. Previously, the findings obtained from surgical lung biopsy were considered the gold standard for ILD diagnosis. The MDM was first recommended to replace histopathology as the gold standard in the 2002 American Thoracic Society (ATS)/European Respiratory Society (ERS) Joint Statement on the Classification of Idiopathic Interstitial Pneumonias (4). This recommendation was re-emphasized in the 2013 update (1) as well as in more recent position statements (5, 6) and clinical practice guidelines for the diagnosis of idiopathic pulmonary fibrosis (7, 8), hypersensitivity pneumonitis (9) and progressive pulmonary fibrosis (8).

Despite strong support for the MDM from expert bodies, there is a paucity of evidence regarding ideal ILD MDM composition. Additionally, reduced access to specialist ILD centers impacts patients and clinicians in many parts of the world. Despite these challenges, it has been demonstrated that a multidisciplinary approach to ILD diagnosis has been widely adopted in routine care settings globally (10). The absence of defined “optimal” features of the ILD MDM has led to a lack of standardization between MDMs, with potential impact on diagnostic integrity and patient outcomes. An evidence-based approach to MDM constituency and processes is clearly a priority, particularly in view of the constantly evolving diagnostic and treatment landscape and the need to integrate new tools and technologies into the paradigm. In this review, we provide an overview of ILD MDMs worldwide, discussing recent developments and highlighting unmet research needs.

### Role of the ILD MDM

Interstitial lung diseases place enormous encumbrance on patients, carers, and health care systems. Many patients with ILD are at risk of progressive fibrotic disease, associated with reduced quality of life and premature mortality. Idiopathic pulmonary fibrosis (IPF), the most common fibrotic ILD is almost universally progressive and up to 40% of non-IPF ILD are also observed to progress (11). Both early recognition of the condition and timely initiation of disease-specific therapy are important determinants of outcomes. Antifibrotic therapy to slow disease progression is now standard of care in IPF (12–15), whereas many non-IPF ILDs respond to treatment with immunosuppressive therapy (16). Recent landmark clinical trials such as INBUILD and SENSCIS have also demonstrated efficacy of antifibrotic therapies in non-IPF progressive fibrosing ILDs (17, 18), but there is much still to learn about disease-specific pathogenesis and targetable pathways.

For these reasons, early and accurate diagnosis is critical. ILD MDMs, involving case discussion between health professionals from different specialties to generate a consensus diagnosis for the patient, aim to maximize available clinical data, expertise and therefore, diagnostic accuracy. Traditionally, the ILD MDM is chaired by a respiratory physician with expertise in ILD. Other important contributors include other respiratory physicians, radiologists and histopathologists with expertise in ILD, rheumatologists and immunologists, ILD nurses, trainees, and other health professionals. Availability of resources will dictate the constitution of an MDM at each site. In addition to generation of a consensus ILD diagnosis, the ILD MDM also functions as a forum to consider patient prognosis and management.

### Evidence for the practice of ILD MDMs

Since its implementation, a multidisciplinary approach has consistently been associated with higher levels of diagnostic confidence, better inter-observer agreement and lower rates of unclassifiable diagnoses—considered surrogate markers for diagnostic accuracy (19, 20). For example, Flaherty et al. (21) demonstrated significant improvements in inter-observer agreement and diagnostic confidence following multidisciplinary discussion between expert clinicians, radiologists and pathologists reviewing 58 cases of suspected idiopathic interstitial pneumonia, compared with each individual MDM participant working separately. These findings were replicated in another European study showing far superior levels of diagnostic agreement between local ILD MDMs and expert radiology and tissue pathology panels compared to individual specialties working apart (22).

An international study of IPF diagnosis by 34 expert ILD physicians and 370 non-expert respiratory physicians showed inter-observer agreement to be higher among expert physicians (Cohen's weighted kappa coefficient [κ*w*] = 0.65, IQR 0.53–0.72) than non-expert physicians (κ*w* = 0.53, IQR 0.41–0.63) and physicians without access to an ILD MDM demonstrated the lowest rate of inter-observer agreement (κ*w* = 0.46, IQR 0.33–0.58). Importantly, association with an academic hospital or university, longer duration of ILD experience and access to an ILD MDM were all independently associated with greater prognostic accuracy of IPF diagnosis, thus demonstrating the clinical benefit of having experienced physicians present at the ILD MDM and training of non-experienced clinicians (23).

An Australian analysis of the clinical impact of the ILD MDM showed that multidisciplinary discussion resulted in a change in diagnosis in 53% of ninety consecutive patients with suspected ILD presenting to two specialist ILD centers (24). Importantly, there was a significant reduction in unclassifiable ILD diagnoses; with 42% of patients initially diagnosed with unclassifiable ILD by their referring physicians, and only 12% remaining unclassifiable following MDM discussion. These findings have been replicated in subsequent larger studies, which have also shown a trend toward greater prognostic separation for an MDM ILD diagnosis compared with pre-MDM individual clinician or radiologist diagnosis (25, 26).

Data that MDMs change therapeutic practice is limited. However, ILD MDMs have been shown to increase recommendations for antifibrotic therapy, steroid-sparing immunosuppressive agents, pulmonary vasodilators, clinical trial participation and supplemental oxygen prescription (24, 27).

## Variability of multidisciplinary meetings around the world

### Inter-MDM heterogeneity

Although this multidisciplinary approach to ILD diagnosis has been widely adopted, significant heterogeneity exists with regards to the structure and processes of ILD MDMs. The inconsistent diagnostic concordance between different MDMs may in-part reflect varying approaches to clinical decision-making or other MDM factors.

The largest multicenter evaluation of diagnostic concordance between different ILD MDMs to date was conducted by Walsh et al. in 2015 (26). Diagnostic concordance between seven ILD MDMs (each consisting of at least one clinician, radiologist, and pathologist) in Europe and the United Kingdom, was evaluated; based on sequential review of 70 patients presenting to an ILD expert center. Inter-MDM for first choice diagnoses overall was only moderate (κ = 0.50). Inter-MDM agreement was better for IPF [κ*w* = 0.71 (IQR 0.64–0.77)] and connective tissue disease-associated ILD (CTD-ILD) [κw = 0.72 (0.68–0.78)]; however, only moderate for non-specific interstitial pneumonia (NSIP) [κw = 0.42 (0.37–0.49)], and fair for hypersensitivity pneumonitis (HP) [κw = 0.29 (0.24–0.40)].

Various reasons have been proposed as to why such discordance exists (19, 20, 28–33). Firstly, the intrinsic heterogeneity of individual ILDs and/or overlapping clinical, radiological, or histopathological features naturally results in difficulty distinguishing specific diagnoses. For example, chronic HP may also be associated with a UIP pattern on high-resolution computed tomography (HRCT), often making it difficult to distinguish from IPF. Secondly, differing approaches to interpretation of international clinical practice guidelines have been observed; with some ILD MDMs adhering more strictly to guidelines and others more likely to assign pragmatic clinical diagnoses with a view to facilitating treatment. Differences between individual physicians, including with regards to training and exposure to ILD logically impact on their diagnostic processes.

Importantly, factors relating to MDM structure, organization and administration, governance and clinical decision-making processes are likely to differ between MDMs. Jo et al. (34) surveyed ten expert ILD centers in Europe, North America and Australia; and identified significant differences in MDM constitution. Specifically, attendance by various specialists such as rheumatologists, quantity and method of data presentation and approach to formulation of diagnosis varied considerably between centers.

### A global perspective of the ILD MDM

An evaluation of 457 centers across 64 countries in Europe, the Asia-Pacific region, North America, South and Latin America, Middle East and Africa performed between 2016 and 2017 *via* electronic questionnaires showed 79.6% held formal ILD MDMs to discuss patient diagnosis and management (10). However, the composition of the MDMs was heterogeneous. For example, centers in lower-income healthcare settings including Brazil, Russia, India, and China were more likely to discuss new cases at an ILD MDM than other countries, however held fewer formal meetings (median 50% compared with 80%). Responses from these countries were more likely to be from academic ILD centers than non-specialist centers, except for India which had a higher proportion of non-academic centers (52.6%).

Centers in high-income countries were more likely to hold meetings at least every 2 weeks (66.8 vs. 53.3%), hold solely face-to-face meetings (83.6 vs. 73.3%), have meetings of between 31 and 60 min duration (52.4 vs. 33.3%) and have at least four disciplines in attendance (60.8 vs. 33.3%) compared with participating centers in lower-middle income countries (10). Although low-income countries were under-represented in this study, it is fair to conclude that variability in MDM processes across the globe is likely at least in part related to reduced access to resources and multidisciplinary expertise in remote and poorer settings (35). A survey of 455 physicians managing IPF patients in Latin America published in 2018 showed that only 27.8% reported access to pathologists, 39.4% to radiologists and a mere 26.9% to multidisciplinary teams (36).

### Diagnosing connective tissue disease-associated ILD (CTD-ILD) at ILD-MDM

Although up to 20% of ILDs are associated with underlying CTDs such as systemic sclerosis, rheumatoid arthritis and idiopathic inflammatory myopathies, rheumatologists and immunologists are not routinely involved in ILD MDMs (37, 38). In fact, an evaluation of global MDM practices showed only 37.1% of survey centers routinely involved a rheumatologist in ILD MDM discussions (10), and rheumatology opinion was otherwise only sought if the referring clinician suspected a systemic autoimmune disease based on their own assessment. The implications of this approach include the potential to miss CTD diagnoses, resulting in delayed institution of immunosuppressive treatments that might reverse disease and prevent irreversible lung fibrosis. Since rheumatologists or immunologists are not always accessible in every setting, it is somewhat reassuring the CTD-ILD is more reliably diagnosed at MDM than some other ILDs such as chronic hypersensitivity pneumonitis (HP) (26).

A recent observational study from a large Italian ILD expert center analyzed consecutive ILD MDM cases with suspected new diagnosis or progression of CTD-ILD, concluding that involvement of a rheumatologist in the MDD resulted in availability of more comprehensive clinical information and improved diagnostic accuracy (37).

Another recent Italian study involving a Delphi survey and additional questionnaires distributed to pulmonologists, rheumatologists and radiologists demonstrated high levels of agreement regarding the importance of a collaborative approach to diagnosis of CTD-ILD (38). Results were used to generate checklists of important “red flag” signs and symptoms suggestive of ILD in CTD patients, as well important “red flag” signs and symptoms suggestive of CTD in undifferentiated ILD patients. Importantly, the Delphi survey also addressed potential methods to improve recognition of CTD-ILD where rheumatologists are not present at the ILD MDM. These included creation of networks and collaborative research efforts between different centers across the country; and development of opportunities for clinicians to participate in multidisciplinary clinics or locoregional ILD MDMs discussing archetypal cases; with the aim of improving evidence-based approach to diagnostic formulation in challenging ILDs (38).

### Expert consensus regarding essential features of the ILD MDM

Despite scarcity of supporting evidence, expert panels have suggested approaches to ILD MDM organization in attempts to provide a standard framework for universal implementation (5). As a minimum standard, these have recommended attendance by at least two respiratory physicians in addition to a radiologist and tissue pathologist, recognizing that this is clearly not feasible in every setting. Other key components, including core data inputs and outputs for each case are outlined in Table 1. Importantly, consensus should be achieved on whether there is a need for invasive testing with lung biopsy for each case (7, 8). Additionally, international consensus guidelines on standardized diagnostic ontology for fibrotic ILDs have also recommended classification of expected disease behavior to help inform patient prognosis, treatment goals and future monitoring strategy (1, 5, 28).

**Table 1:** Key data inputs and outputs for each case discussed at the ILD MDM, based on international expert consensus guidelines.

** *Core data to be presented for each case:* **
1. Comprehensive clinical history and physical examination findings, including:
• Smoking history
• Occupational, environmental, drug or other exposures known to be associated with hypersensitivity pneumonitis or occupational lung disease^a^
• Family history of pulmonary fibrosis or autoimmune disease
• Symptoms and signs suggestive of underlying CTD
2. Investigations, including:
• Autoimmune serology – including at least antinuclear antibody (ANA), anti-cyclic citrullinated peptide (anti-CCP), rheumatoid factor (RF).Other autoimmune serology including an extended myositis panel is considered on a case-by-case basis according to symptoms and signs^b^
• Detailed pulmonary function testing results
• High-resolution CT scan^c^
** *Core outputs to be documented for each case:* **
1. Consensus ILD diagnosis
2. Degree of diagnostic confidence^d^
3. Any differential diagnoses
4. Expected disease behavior^d^
5. Suggested management plan, including whether there is a need for additional testing with bronchoalveolar lavage (BAL), transbronchial lung cryobiopsy (TBLC) or surgical lung biopsy

^*a*^*See References* *(**1*, *3*–*8*, *39**)*.

^*b*^*Including antibodies to extractable nuclear antigens (anti-Ro [SS-A], anti-La [SS-B], anti-Smith, antiribonucleoprotein [anti-RNP], antitopoisomerase [Scl-70], anti-Jo-1), anti-double stranded DNA antibodies (anti-dsDNA), anti-tRNA synthetase antibodies (Jo-1, PL-7, PL-12, EJ, OJ, KS, Zo, tRS), extended myositis panel (including antibodies to Ro-52, Ku, Mi-2, TIF1-*γ*, PM-Scl, MDA-5, NXP2, SAE1) and anti-neutrophil cytoplasmic antibody (ANCA)* *(**7*, *40**)*.

^*c*^*Performed according to technical standards outlined in ATS/ERS/JRS/ALAT Clinical Practice Guideline for Diagnosis of IPF* *(**7**)*.

^*d*^*According to internationally standardized diagnostic ontology for fibrotic ILD* *(**28**)*.

An example of a standardized framework for the ILD MDM is the 2017 Thoracic Society of Australia and New Zealand and the Lung Foundation Australia Position Statement on the ILD MDM and accompanying ILD toolkit, consisting of practical material designed to aid in the presentation and discussion of cases at ILD MDMs (5, 41)^1^ This included suggested template formats for presentation of case data, suggested sequence for case discussion and recommended standard nomenclature. The clinical impact of this toolkit is currently unknown.

It is also worth noting the proliferation of interstitial lung disease registries worldwide in the last 20 years, such as the Australasian ILD Registry (AILDR) (42), the Pulmonary Fibrosis Foundation Patient Registry (PFF-PR) (43), Canadian Registry for Pulmonary Fibrosis (CARE-PF) (44), and many others, including in developing countries (45–47). These important research repositories have provided a standardized platform for documentation of ILD MDM outcomes by participating centers, which will help to facilitate future collaborative research into ILD diagnostic and treatment pathways.

Worldwide standardization of the ILD MDM will require an understanding of factors contributing to diagnostic heterogeneity as well as consensus regarding the purpose and desired outcomes of multidisciplinary discussion. Recently, Teoh et al. (48) conducted informative research into the key components of an ILD MDM, through semi-structured interviews and subsequent Delphi surveys among ILD physicians across nine countries.

Experts strongly agreed upon five essential features for an ILD MDM, including: 1) the need for at least one radiologist, 2) high-quality HRCT images for each case, 3) technological infrastructure enabling real-time viewing of CT scans, 4) a quiet environment enabling uninterrupted, free-flowing discussion and 5) a standardized template for documentation of outputs from each case discussion. Additionally, experts agreed upon several other desirable components as outlined in Table 2. The need for validation of MDM processes following the genesis of robust evidence was identified as a priority.

**Table 2:** Essential and desirable features of the ideal ILD MDM based on expert consensus^a^.

**Who should attend?**
• Attendees should include:
° At least two respiratory physicians
° At least one radiologist
° At least one tissue pathologist
° Specialist trainees and fellows, with the purpose of gaining education and expertise in ILD
° External physicians, either in-person or *via* videoconferencing
• At least one participant should have >5 years ILD experience and ideally >1 member from each discipline should be present
**Where should it occur?**
• Quiet setting - to enable uninterrupted discussion and encourage participation
**When should it happen?**
• Regularly scheduled meeting date
**What technology is recommended?**
• Visual projection system allowing real time viewing of HRCT images
**How should the meeting be organized?**
• A chairperson should be nominated to moderate the discussion
• A meeting coordinator should ensure all relevant information is available prior to each MDM
• Strategies should exist to prioritize urgent cases for discussion
• Regular review of ILD MDM policies and protocols should occur
**What information should be available for each case?**
• Thorough clinical history, good quality HRCT scan and autoimmune serology
• Pulmonary function testing including at least spirometry and DLCO
• Histopathology images for patients who have undergone lung tissue sampling
• Tissue pathologists should review tissue samples prior to ILD MDM
**What information should be documented following each case discussion?**
• Consensus diagnosis
• Diagnostic confidence, with acknowledgment that provisional or unclassifiable diagnoses may require re-presentation when new information available
• Differential diagnoses
• Initial treatment and management recommendations
**How should information be presented and documented?**
• Case information should be collated prior to each MDM for display using a standardized template format
• Results of each case discussion should be documented using a standardized template
• Processes should exist to communicate outputs to referring physician and any other relevant stakeholders
**How should the MDM approach clinical decision-making?**
• Adherence to current standardized diagnostic guidelines^b^
• Standardized research terminologies, for example “idiopathic pneumonia with autoimmune features” (IPAF) to considered as consensus diagnosis
How will MDMs ensure they remain compliant with***“*****best practice*****”*** **in the future?**
• Fulfillment of minimum number of case discussions annually
• Annual revision process to compare ILD MDMs to internationally established benchmarking guidelines (once these have been published)
• Regular self-assessment using international case database

^*a*^*Adapted from Teoh et al*. *(**48**)*.

^*b*^*Including ATS/ERS/JRS/ALAT Clinical Practice Guideline on Idiopathic Pulmonary Fibrosis and Progressive Pulmonary Fibrosis in Adults and ATS/JRS/ALAT Clinical Practice Guideline on Diagnosis of Hypersensitivity Pneumonitis in Adults* *(**7*, *8**)*.

Many questions remain. Notably, there was no consensus agreement upon the need for attendees from other specialty types such as rheumatology or immunology; and although a histopathologist was felt to be “highly desirable”, their presence was not considered essential. This likely reflects limited access, particularly in smaller and more remote centers. The authors also noted that viability is a concern, with ongoing increases in the workload of ILD MDM clinicians, radiologists, and pathologists, limiting time and resources available to dedicate to consideration of each available case. Of note, the pathologist is a key contributor in only a small fraction of cases where a biopsy has been performed. Additionally, in this modern era, lack of onsite pathology does not preclude involvement in the MDM as tissue pathologists can participate virtually even if a center does not have an onsite pathologist.

Interestingly, ILD experts agreed that research terminologies such as IPAF could also be documented as present by consensus at MDM. Although this term has not been validated as a distinct diagnostic entity, its use may be appropriate where the ILD MDM's favored diagnosis is suspected CTD-ILD, rather than idiopathic interstitial pneumonia, but the patient doesn't satisfy CTD diagnostic criteria. However, further studies using refined IPAF criteria are likely required before IPAF can be considered a distinct diagnosis and to inform evidence-based management of affected patients.

The ideal format of an ILD MDM also remains uncertain—whether meeting entirely face-to-face, virtually *via* videoconferencing or web-based platforms, or a hybrid model, is best. Nonetheless, these consensus agreements on the ideal features of an ILD MDM are fundamental in informing future research into MDM standardization.

## Recent developments

Recent scientific and technological developments have had significant impact on both the inputs and outputs of the ILD MDM. Expanded therapeutic options and an increasing array of clinical trials have added to the complexities of management. Furthermore, the global pandemic has necessarily transformed the essence of human interaction, particularly in the healthcare setting. Some key recent influences on the ILD MDM are considered below.

### Availability of antifibrotic therapies for IPF and progressive pulmonary fibrosis (PPF)

Evolution of clinical practice guidelines for diagnosis of ILDs and the availability of the antifibrotic drugs nintedanib and pirfenidone have been associated with increased frequency and complexity of referrals to ILD specialist centers for MDM consideration (49). Changes in patterns of MDM behavior have also been observed, particularly in parts of the world where an MDM diagnosis is required by regulatory prescribing bodies to access antifibrotic therapy.

In these settings, it can be tempting to label patients as having IPF or PPF despite an alternative leading differential diagnosis in order to overcome the limitations of regulatory prescribing. Although this is a pragmatic strategy, it is worth acknowledging the risks associated with such practice. With respect to the “PPF” (or progressive fibrosing ILD “PF-ILD”) label in centers where antifibrotics are available for this indication, “lumping” a heterogeneous group of patients together, rather than “splitting” into specific diagnostic groups risks limiting opportunities to identify inflammation-driven disease activity or other causes of deterioration. International guidelines emphasize the treatment for PPF must be agnostic to the underlying condition and all efforts must be made to procure and treat a leading diagnosis before labeling it as progressive (8).

### Emergence of transbronchial lung cryobiopsy (TBLC)

TBLC has recently been demonstrated in both clinical trials and meta-analyses to be a reliable method for lung tissue sampling for histopathologic assessment and MDM diagnosis in ILD; a less invasive procedure with lower rates of complications such as pneumothorax and airway bleeding compared with traditional surgical lung biopsy although the quality of evidence is low (50–52). The landmark COLDICE trial demonstrated high levels of diagnostic concordance between TBLC and surgical lung biopsy ILD multidisciplinary discussions; and TBLC MDM diagnoses made with high confidence were even more reliable, showing excellent (95%) concordance with surgical lung biopsy MDM diagnosis (52).

The recently updated ATS/ERS/JRS/ALAT clinical practice guideline for IPF and PPF (8) includes a conditional recommendation to regard TBLC as an acceptable alternative to surgical lung biopsy in centers with appropriate expertise. The 2022 ERS Guidelines on TBLC in the diagnosis of interstitial lung diseases recommend TBLC as either a replacement first test in patients considered eligible for surgical lung biopsy (SLB); or as an option for patients considered unsuitable for SLB (53). As such, consideration of TBLC is likely to become more widely adopted into the ILD MDM diagnostic paradigm.

### Novel diagnostic tools

There has been significant recent research interest into novel tools to increase diagnostic yield and accuracy in ILD diagnosis, including the use of genomic classifier testing (54).

Importantly, there is a growing understanding of pathogenic mutations linked to an inherited risk of pulmonary fibrosis. Mutations in telomere-related genes such as telomerase reverse transcriptase (TERT) have been identified in up to one-quarter of familial pulmonary fibrosis patients and have been associated with the “short telomere syndrome”, predisposing affected individuals to progressive ILD, premature hair graying, cryptogenic liver cirrhosis, hematological abnormalities, and reduced survival (55, 56). Despite variable pulmonary fibrosis phenotypes amongst patients with telomere-related gene mutations, affected individuals have been shown to experience consistently progressive disease, more rapid lung function decline and worse transplant-free survival than unaffected individuals (56). Rare variants in genes affecting surfactant metabolism in familial pulmonary fibrosis patients are also associated with an increased risk of lung adenocarcinoma (56). In addition to the prognostic implications, short telomere length has been associated with worse outcomes in patients treated with immunosuppressive medications and similar adverse outcomes have also been shown in patients with sporadic pathogenic mutations in telomere-related genes without a family history (56).

Integration of genetic data into the ILD MDM is yet to be validated, yet research is underway. A recent international survey including 352 respiratory physicians demonstrated support for the use of genetic testing in ILD, predominantly in view of its impact on diagnostic investigation approach and patient treatment, however 88% identified a need for more information on its role and interpretation of results (57).

Currently, genomic testing might be considered within the MDM for patients with a family history or atypical disease presentation, to help predict expected disease behavior and guide management discussions. For example, patients with inherited or sporadic ILD-associated genetic mutations might be recommended for earlier consideration of lung transplant referral, and avoidance or caution with immunosuppressive therapy. It could also be recommended with a view to potential clinical trial participation, such as current phase II studies into the use of the androgen danazol for treatment of familial pulmonary fibrosis associated with short telomere length (58). Additionally, genetic testing results have important implications for screening of family members of ILD patients. It is likely that genetic data is close to being implemented as an important consideration within the ILD MDM and will be used more widely in the future as access to testing and knowledge improves, and evidence-based guidelines become available.

The Envisia^TM^ genomic classifier, which employs a machine learning algorithm developed to classify usual interstitial pneumonia (UIP) vs. non-UIP ILD patterns, uses bulk RNA-sequencing data obtained from high throughput sequencing of exome-enriched RNA extracted from transbronchial lung biopsies or TBLC (59). Numerous studies have demonstrated high specificity performance of the classifier to predict UIP in fibrotic ILD (60–63), however its sensitivity for UIP is only 68% and so many cases will still require lung tissue sampling (54). Kheir et al. (64) assessed the impact of sequentially presented data from TBLC and genomic classifier results on the diagnostic confidence of ILD MDMs. The classifier increased diagnostic confidence when added to TBLC for patients with a probable UIP pattern; although did not impact as significantly on the proportion of high-confidence diagnoses as did the addition of the TBLC result. The quality of available evidence was rated as low (54). As such, recent guidelines have made no recommendations either for or against the use of genomic classifier testing in clinical practice (8).

Other novel diagnostic methods with significant potential include the use of artificial intelligence and computer-based deep learning algorithms for the assessment of HRCT images and digital histology slides (65–67). As with genomic biomarkers, future controlled studies are required before these techniques are ready for widespread adoption into clinical practice and integration into the ILD MDM process.

### Use of video and web-based technologies during the COVID-19 pandemic

The COVID-19 pandemic has dramatically impacted ILD services globally, with substantial disruption to usual clinician-patient interactions, access to diagnostic procedures and testing and face-to-face ILD MDMs. Due to social distancing requirements and widespread illness, many centers have adopted either entirely virtual MDMs or hybrid in-person and virtual MDMs, with some participants present *via* teleconferencing (68). Some limitations of this format of course exist, including potential technical issues and reduced participation by some attendees due to inherent differences between individuals and unfamiliarity or discomfort with the virtual format. However, it has generally been useful to allow continuation of ILD MDM service provision at both ILD expert centers and remote centers during the pandemic. Future studies are underway to inform the utility of virtual MDMs compared with conventional MDMs.

An innovative approach involving the use of a national cloud-based database integrating clinical, radiological and pathological data for ILD patients along with a web-based ILD MDM system was explored in Japan (69). Web-based MDMs were conducted with attendant pulmonologists, radiologists and pathologists for 465 cases of biopsy-proven IIPs. Web-based multidisciplinary discussion resulted in a change in diagnosis for 47% of patients, and improved prognostic discrimination between the pre-MDD and MDD diagnoses. Importantly, 5% of patients were diagnosed with non-idiopathic ILDs by MDD; and a substantial proportion of patients were identified as fulfilling IPAF criteria; with meaningful implications for patient management (69). Although the apparent feasibility of this system is promising, particularly in settings with limited access to ILD expertise, non-real time discussion of retrospective data by clinicians who have not physically reviewed the ILD patient likely limits the availability of important clinical data necessary for a precise diagnosis.

## Suggested approach

**Figure 1:**
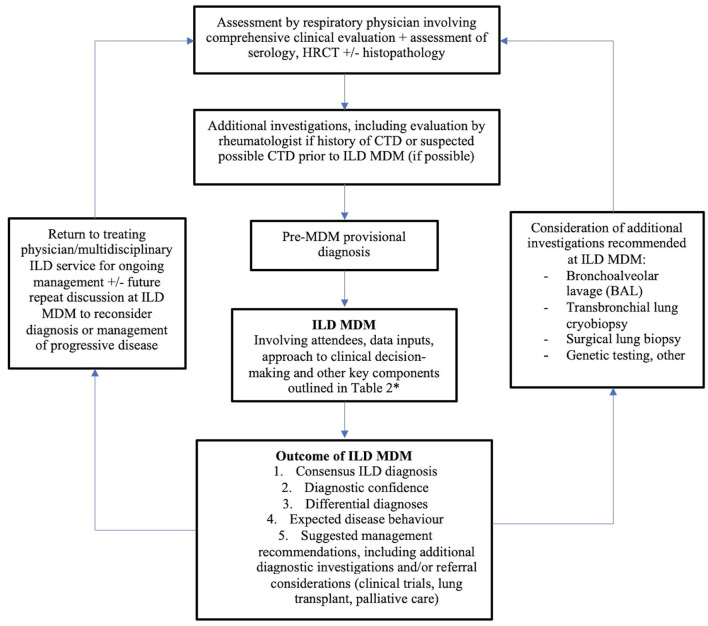
The role of the ILD MDM in diagnosis and ongoing clinical care^a^. ^a^Adapted from Prasad et al. (5). *Plus attendance by rheumatologist and/or immunologist where available.

Until additional evidence exists regarding the optimal ILD MDM, we suggest a multidisciplinary approach to ILD diagnosis as outlined in Figure 1; an approach adapted from the TSANZ/LFA 2017 position statement (5) and supported by preliminary data. Structure and coordination of the MDM should be based on current international expert consensus, as outlined in Table 2. Although the presence of at least one radiologist in addition to respiratory physicians is considered essential, other specialists' attendance will be dictated by availability. Ultimately, the ideal ILD MDM is comprised of a cohort of interested, enthusiastic individuals, since it should also be a learning environment in addition to its other functions. Uptake of the ILD MDM is essential to address current needs as well as train future ILD clinicians by propagating knowledge and expertise.

The treating physician should consider re-presentation of patient cases at the ILD where disease evolution or results of additional investigations are likely to result in a change in diagnosis; or to obtain consensus agreement upon management of progressive disease.

## Conclusions and future directions

The MDM has an integral role in the diagnosis of ILD, with considerable implications for patient management and future outcomes. Therefore, it is critical that the optimal structure, processes, and governance of the ILD MDM are optimized and validated; with a view to standardization of the ILD MDM worldwide. Additional future research priorities will include the integration of novel diagnostic techniques such as genetic and molecular biomarker data for use within the ILD MDM, including consideration of their implication for personalized treatment approaches for ILD patients. The role of regional, national, or transnational ILD MDMs in order to standardize and improve ILD expertise and enhance access to multidisciplinary discussion should also be considered.

## Author contributions

LG wrote the manuscript, with input from LT and TC. All authors reviewed the manuscript and agree with regard to the contents.

## Funding

This research is supported by a Lung Foundation Australia scholarship, with matched funding provided by the University of Sydney (Lung Foundation Australia/Diana Cox Idiopathic Pulmonary Fibrosis Ph.D. Scholarship 2019).

## Conflict of interest

LG has received travel and conference support from Boehringer Ingelheim. LT has provided paid consultancy for Erbe Elektromedezin and Boehringer Ingelheim. TC has received grant support, consultancy fees, and speaking honoraria from Boehringer Ingelheim and Hoffman-La Roche, consultancy fees from Bristol Myers Squibb; grant support from Biogen, and provides consultancy for DevPro and Ad Alta.

## Publisher's note

All claims expressed in this article are solely those of the authors and do not necessarily represent those of their affiliated organizations, or those of the publisher, the editors and the reviewers. Any product that may be evaluated in this article, or claim that may be made by its manufacturer, is not guaranteed or endorsed by the publisher.

## Footnotes

^1^ https://lungfoundation.com.au/resources/ild-mdm-toolkit-guide/
